# Current Advances in RNA Therapeutics for Human Diseases

**DOI:** 10.3390/ijms23052736

**Published:** 2022-03-01

**Authors:** Hannah Zogg, Rajan Singh, Seungil Ro

**Affiliations:** Department of Physiology and Cell Biology, Reno School of Medicine, University of Nevada, 1664 North Virginia Street, Reno, NV 89557, USA; hannahphillips@unr.edu (H.Z.); rajans@med.unr.edu (R.S.)

**Keywords:** RNA therapeutics, non-coding RNA, miRNA, ASO, siRNA, aptamer, mRNA, cancer, diabetes, nanoparticles

## Abstract

Following the discovery of nucleic acids by Friedrich Miescher in 1868, DNA and RNA were recognized as the genetic code containing the necessary information for proper cell functioning. In the years following these discoveries, vast knowledge of the seemingly endless roles of RNA have become better understood. Additionally, many new types of RNAs were discovered that seemed to have no coding properties (non-coding RNAs), such as microRNAs (miRNAs). The discovery of these new RNAs created a new avenue for treating various human diseases. However, RNA is relatively unstable and is degraded fairly rapidly once administered; this has led to the development of novel delivery mechanisms, such as nanoparticles to increase stability as well as to prevent off-target effects of these molecules. Current advances in RNA-based therapies have substantial promise in treating and preventing many human diseases and disorders through fixing the pathology instead of merely treating the symptomology similarly to traditional therapeutics. Although many RNA therapeutics have made it to clinical trials, only a few have been FDA approved thus far. Additionally, the results of clinical trials for RNA therapeutics have been ambivalent to date, with some studies demonstrating potent efficacy, whereas others have limited effectiveness and/or toxicity. Momentum is building in the clinic for RNA therapeutics; future clinical care of human diseases will likely comprise promising RNA therapeutics. This review focuses on the current advances of RNA therapeutics and addresses current challenges with their development.

## 1. Introduction

For years it was believed that DNA was transcribed into RNA and that this RNA (messenger RNA) was then translated into a protein; however, this all became more complicated when RNA interference (RNAi) was discovered [1,2]. RNAi is a conserved biological process in which there is a repression of gene expression caused by small RNAs (i.e., microRNAs (miRNAs) and synthetic small interfering RNAs (siRNAs)) interacting with protein complexes, such as the RNA-induced silencing complex (RISC) [3,4]. Once bound, the small RNAs can then bind to their respective target mRNA in a sequence-specific manner to either stop translation or target the mRNA for degradation [5]. This discovery led to a huge increase in research focused on treatments for diseases that would exploit RNAi instead of traditional treatments focused on utilizing small molecules and proteins.

Most drugs currently on the market are either small molecules or proteins. Small molecule-based drugs are commonly competitive inhibitors of their target proteins, while protein-based drugs are commonly used to bind to target proteins, replace non-functional target proteins, or supplement for an inadequate amount of a target protein [6,7,8]. A serious issue with protein-based drugs is that most proteins are too large to enter their target cells and therefore are only effective when their target molecule is extracellular or excreted [9]. While small molecule- and protein-based drugs have been found to be effective in many cases, there is still a plethora of diseases that are unable to be treated using either small molecules or proteins. For example, many diabetic patients develop insulin resistance, and supplementing additional insulin is no longer effective in lowering their blood glucose levels. However, RNA-based treatments may have therapeutic potential for diseases, such as diabetes, cancer, and Huntington’s disease [2,10,11,12,13]. RNA therapies may provide better treatment options to target the pathophysiological mechanisms of these disorders, which may lead to better outcomes for patients. Additionally, many RNA therapies have already been approved by the United States Food and Drug Administration (FDA), with more therapies in various phases of clinical trials demonstrating the validity of such RNA therapies for various diseases. This review will cover the mechanisms of RNA therapy design, current FDA-approved RNA therapies, RNA therapies in clinical trials, and RNAs with clinical potential for treating patients suffering from various conditions.

## 2. RNA Therapeutics

**Figure 1:**
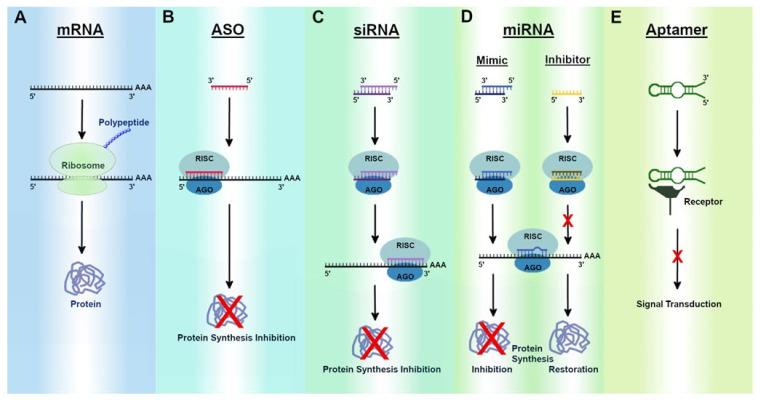
Schematic of RNA therapeutic approaches. (**A**) Ribosomes translate mature mRNAs into proteins, the building blocks for life. (**B**) ASOs are small single-stranded RNA molecules that have exact complementarity to a target mRNA. Once bound, they induce post-transcriptional gene silencing by preventing translation of the mRNA. (**C**) siRNAs are small double-stranded RNA molecules that have exact complementarity to a target mRNA. Once associated with the RISC complex, it binds to its target mRNA and induces gene silencing by preventing translation of the mRNA. (**D**) miRNA mimics are small double-stranded RNA molecules that associate with and guide the RISC complex to its target mRNA. The mimic will bind with imperfect complementarity to its target mRNA, and translation will be blocked or the mRNA will be degraded leading to gene silencing. miRNA inhibitors are small single-stranded RNAs that bind to and suppress their target miRNA. This results in restored mRNA translation. (**E**) Aptamers are RNA, DNA, or RNA/DNA hybrids that form tertiary structures and bind to a target molecule, either suppressing or enhancing the pathway that the target molecule is involved in.

RNAs as therapeutic agents have been vastly studied over the past few decades, as they are more cost-effective and easier to develop than traditional small molecule- or protein-based therapeutics [2]. As of now, there are five different categories of RNA therapeutics (Figure 1): (1) messenger RNAs (mRNAs) that encode for proteins, (2) antisense oligonucleotides (ASOs) that are small (~15–25 nucleotides) single-stranded RNAs (or DNAs, but for the purposes of this review, we focus only on RNA ASOs) that can either promote or repress their targets expression, (3) small interfering RNAs (siRNAs) that are similar to ASOs in size; however, they are double-stranded and primarily cause translational repression of their target protein, (4) microRNAs (miRNAs) that are small RNAs that can either inhibit protein synthesis when they bind to an mRNA target (miRNA mimics) or free up mRNA by binding to the miRNA that represses the translation of that particular mRNA (miRNA inhibitors), and (5) aptamers that are short single-stranded nucleic acids that form secondary and tertiary structures and interact with a specific enzyme or molecule and therefore can promote or inhibit many different molecular pathways. Within these five categories, there are a handful of already US FDA-approved medications (Table 1), many of which are in clinical trials (Table 2), and an even more that have been found to have possible therapeutic potential but are not yet in clinical trials. While RNA therapeutics seem to have immense potential, there are many hurdles that need to be surmounted when they are developed clinically to make them effective potential treatment options.

Before RNAs were used therapeutically, there were many challenges that had to be overcome in order to make them feasible treatment options for human diseases. For example, nucleic acids are negatively charged and do not passively cross the hydrophobic lipid barrier of the cell. Further, exogenous RNAs are degraded rapidly by RNases once they are injected into the host. Finally, some exogenous RNAs cause an immune response that hampers the translation of the target protein or causes the development of a toxic cell environment. Luckily, scientists over the past couple of decades have substantially overcome these barriers with the use of many unique delivery methods, such as nanoparticles that protect the RNA and enable cell-specific delivery of the therapeutic agent.

### 2.1. mRNA Therapeutics and Functional Implications

mRNA is coding RNA that is transcribed using genomic DNA as a template and serves to encode proteins [68]. mRNAs are typically around 2 kb in length and characteristically contain a 5′ cap, 5′ UTR, coding region, 3′ UTR, and poly(A) tail (Figure 1A) [69]. mRNAs are excellent candidates for the treatment of diseases with a known genetic component. Traditionally, mRNAs have been used for replacement therapy when diseases are caused by a lack of expression of a particular protein [70]. Additionally, CRISPR–Cas-based mRNA therapies can be used to repair DNA mutations that cause non-functional downstream products [71]. In 1990, Wolff et al., were one of the first groups to induce expression of a protein in vivo through the injection of a synthetic mRNA that encoded beta-galactosidase, chloramphenicol, or luciferase into the skeletal muscle of mice [72]. Further, Jirikowski et al., used mRNA to treat diabetes insipidus in Brattleboro rats that have reduced levels of vasopressin [73]. Synthetic or in vitro exogenous vasopressin mRNA was able to temporarily reverse diabetes insipidus when injected in these rats. These initial reports implicated mRNAs as possible therapeutic options for treating/preventing human diseases.

#### mRNA Vaccines

mRNAs that encode either adjuvants or antigens have also been proposed as possible vaccine candidates that may be able to prevent many diseases. In 1993, Martinon et al., reported that there was induction of anti-influenza cytotoxic T lymphocytes following immunization with liposome-complexed mRNA-encoding influenza virus nucleoproteins in a murine model [74]. This was the first report highlighting the possible use of mRNAs as vaccines to prevent many infectious diseases. While this study was conducted decades ago, mRNA vaccines have become more common in recent years, especially since the COVID-19 pandemic. Further, this pandemic has also highlighted the speediness of the development of mRNA vaccines when compared to many of the traditional vaccine technologies. The first vaccines to receive emergency use authorization from the FDA were mRNA vaccines.

Currently, there are two mRNA vaccines that are FDA approved and one that is in clinical trials. The first mRNA vaccine approved by the FDA was BNT162b2 [31]. This vaccine was produced in collaboration between Pfizer and BioNTech to create immunogenicity and antibody response to SARS-CoV-2, which causes COVID-19. This vaccine candidate was clinically tested in Germany and the US and was found to significantly decrease the risk of contracting COVID-19. BNT162b2 encodes for the full-length membrane-anchored spike (S) protein that includes two minor mutations to increase conformational stability. The second FDA-approved mRNA vaccine was mRNA-1273, which encodes the perfusion stabilized S protein of SARS-CoV-2 as well as the S1–S2 cleavage site [32]. Similar to BNT162b2, the mRNA-1273 vaccine was manufactured by Moderna to prevent the contraction of COVID-19. Both FDA-approved mRNA COVID-19 vaccines include 1-methyl-pseudouridine to hamper innate immune sensing, while also increasing the translational ability of the mRNA. Additionally, they both are encapsulated by lipid nanoparticles. Further, both vaccines have been shown to provide significant immunity against SARS-CoV-2 infection, while also maintaining high safety standards. The exceptional speed at which both vaccines were developed should be noted. To date, no other vaccine has been developed with such swiftness while also maintaining efficacy. This highlights the clinical advantage of using RNA-based vaccines in treating deadly diseases.

Along with the two FDA-approved mRNA vaccines, there are two additional vaccine candidates. The first is CVnCoV, which is currently in Phase III clinical trials [52]. This vaccine candidate is produced by CureVac AG and is a chemically unmodified mRNA that encodes the full-length S protein of SARS-CoV-2 and utilizes the RNActive mRNA vaccine platform [52]. CVnCoV induces a robust immune response and provides immunity against SARS-CoV-2 infection. The second vaccine candidate is CV7202 and is in Phase I clinical trials for rabies prevention [75]. This vaccine is composed of rabies virus glycoprotein mRNA to induce a rabies neutralizing antibody response. Additionally, there are many mRNA vaccine candidates that are currently in pre-clinical trials or that have pre-clinical potential [74,76,77,78,79,80,81].

To date, there are several mRNA drug candidates in clinical trials for various human diseases: (1) AZD8601 is a vascular endothelial growth factor A (VEGF-A) drug candidate manufactured by AstraZeneca for ischemic heart disease [53]. VEGF-A has been promising in preclinical trials for increased vessel collateralization [82]. This study suggested that VEGF-A might be a good drug candidate for treating ischemic heart disease. However, studies have found that treatment with VEGF-A DNA or viral vectors was safe but not effective in treating ischemic heart disease [83,84]. Therefore, it was hypothesized that VEGF-A mRNA drug candidates may be a more efficacious way of treating this disease. AZD8601 is currently being evaluated in Phase II clinical trials. (2) MRT5005 is manufactured by Translate Bio and is currently in Phase I/II clinical trials for the treatment of cystic fibrosis (CF) lung disease [54]. CF is caused by mutations in the CF transmembrane conductance regulator (*CFTR*) gene. This mutation leads to the buildup of mucous in many organs, especially the lungs [85]. MRT5005 encodes for CFTR and can be delivered through nebulization. (3) mRNA-3704 is in Phase I/II clinical trials for the treatment of methylmalonic aciduria and is manufactured by Moderna [55,56]. Methylmalonic aciduria is a life-threatening genetic disorder in which there is an inability to break down certain proteins and fats, causing a buildup of methylmalonic acid [86]. This disorder is caused by a plethora of mutations in the methylmalonyl-CoA mutase gene. mRNA-3704 encodes for a fully functional methylmalonyl-CoA mutase enabling the breakdown of proteins and fats that were previously unable to be broken down [55,56]. (4) BNT111, manufactured by BioNTech SE, is currently in dose-escalation Phase I clinical trials for the treatment of advanced melanoma [57]. This drug targets NY-ESO-1, MAGEA3, tyrosinase, and TPTE, which are tumor-associated antigens predominantly found in melanoma. This therefore should cause an immune response leading to the destruction of tumor cells. There is also a plethora of other mRNA drug candidates in the preclinical pipeline [87,88,89,90,91].

### 2.2. ASO Therapeutics and Functional Implications

ASOs are synthetic small single-stranded nucleic acid sequences composed of RNA, DNA, or RNA-DNA heteroduplexes that are typically 8–50 bp long [92]. The study of ASOs began in the late 1970s when it was found that synthesized oligonucleotides were able to inhibit Rous sarcoma virus replication [93]. This was achieved through viral protein translation inhibition due to the binding of the synthesized complementary oligonucleotide sequence to the viral 35S mRNA. This study paved the way for ASO-based therapeutics.

Since their discovery, there has been a plethora of other mechanisms of action for ASOs. While there are many different ASOs, they all belong to one of two major categories: RNase H dependent ASOs and RNase H independent/steric block ASOs [94]. RNase H is an endogenous enzyme that catalyzes the degradation of RNA that is part of an RNA–DNA heteroduplex. Therefore, ASOs composed of DNA or both RNA and DNA (gapmers) typically belong to the RNase H dependent category. Once they bind their complementary target mRNA strand, RNase H recognizes the DNA–RNA heteroduplex and catalyzes the degradation of the mRNA. This leads to downregulation of the target mRNA, which can be a useful tool in therapeutic approaches for the treatment of diseases caused by overexpression of certain genes [95], such as homozygous familial hypercholesterolemia [15].

RNase H independent/steric block ASOs are typically composed of only RNA and act by binding directly to the target pre-mRNA or mature mRNA to cause inhibition of mRNA translation (Figure 1B), alternative splicing, promotion of mRNA translation, or alternative polyadenylation. When the ASO binds to the pre-mRNA at a splice recognition site, it will cause alternative splicing of the target RNA. This approach can be extremely useful for the treatment of disorders that are caused by mutations that can be avoided by selective alternative splicing [96]. In general, this type of ASO can also lead to increased expression of the target protein [97]; however, they can also be used to cause exon skipping in order to block translation of the target mRNA [98]. This approach is also known as splice corruption. ASOs have also been known to bind regions of the mRNA, such as the translation initiation codon, to inhibit translation of the mRNA [99]. Further, ASOs that bind regulatory regions upstream of the open reading frame (ORF) have been shown to promote target translation [100]. This is caused by the blocking of regulatory regions typically responsible for translational suppression. ASOs have also been shown to cause alternative polyadenylation by blocking certain polyadenylation sites [101]. This commonly leads to shorter transcripts that contain less destabilized segments, typically leading to increased stability of the mRNA. The many molecular mechanisms of ASOs have enabled their use for many different therapeutic approaches to treat human diseases.

#### 2.2.1. FDA-Approved ASOs

The first FDA-approved ASO was fomivirsen, which is also known as Vitravene. Formivirsen was developed to treat patients with cytomegalovirus (CMV) retinitis, a serious viral eye infection [102]. This ASO therapeutic is composed of 21 phosphorothioate oligodeoxynucleotides that target CMV immediate-early-2 mRNA, which is essential for viral replication [103]. Clinical trials have shown that injections of this drug into the vitreous humor delayed disease progression when compared to untreated controls. Successful clinical trials led to fomivirsen obtaining FDA approval in 1999 [102]. However, disease progression was still inevitable. This, along with the discovery of anti-retroviral therapies, made this medication less pertinent, and it was eventually taken off the market in 2006.

Mipomersen was approved by the FDA in 2013 for the treatment of homozygous familial hypercholesterolemia [104]. This genetic disorder is characterized by increased levels of low-density lipoprotein (LDL) cholesterol due to decreased liver uptake of plasma LDL [105]. This condition leads to premature cardiovascular disease even when treated with previously established lipid-lowering therapies, such as statin therapy [106]. However, treatment with mipomersen, a gapmer 20 oligonucleotides long that targets and reduces expression of the ApoB mRNA, led to significantly reduced LDL levels [15]. ApoB is an essential protein for the clearance of LDL and aids in the production of low-density lipoprotein (VLDL), which is a precursor of LDL [106]. Therefore, reduction of ApoB likely leads to the reduced plasma levels of LDL following mipomersen treatment.

In 2016, nusinersen was approved by the FDA for the treatment of spinal muscular atrophy (SMA) [107]. This disorder is caused by deletions or mutations in the survival motor neuron 1 (*SMN1*) gene. This leads to inadequate SMN protein expression, causing weakness and atrophy of skeletal and respiratory muscles [108]. Nusinersen modulates splicing of *SMN2*, which varies only from *SMN1* in that it undergoes alternative splicing and excludes exon 7 [16]. This exclusion results in a truncated protein that only has 5% to 10% functionality. However, nusinersen regulates alternative splicing such that exon 7 is included, resulting in fully functional SMN leading to improved motor function in patients with SMA.

Eteplirsen was approved by the FDA in 2016 for the treatment of Duchenne muscular dystrophy (DMD) [109]. Mutations in the DMD gene encoding the dystrophin protein leads to the development of DMD [110]. The most common mutation resulting in DMD is located in exon 51; therefore, eteplirsen (a 30-mer ASO) targets exon 51 of the DMD gene, causing this exon to be excluded during alternative splicing [17]. This prevents frame shift mutations that lead to the production of non-functional dystrophin. The resulting dystrophin protein is slightly shorter than its wild-type counterpart but maintains its functionality [111].

Inotersen received FDA approval in 2018 for the treatment of familial amyloid polyneuropathy. This disorder is caused by autosomal dominant mutations in the transthyretin (*TTR*) gene. These mutations disrupt the TTR tetramer leading to aggregation of TTR monomers into amyloid deposits throughout the body [112]. In order to combat the buildup of TTR, inotersen targets the 3′ UTR of the *TTR* mRNA, preventing the production of TTR, thus inhibiting disease progression [113]. Clinical trials demonstrated the efficacy and safety of inotersen in the reduction of circulating TTR levels [18].

Another ASO that has been FDA approved for the treatment of DMD is golodirsen. This drug behaves similarly to eteplirsen, in that it enables exon skipping; however, it leads to the exclusion of exon 53 instead of exon 51 [114]. This results in functional dystrophin and improves symptomology of patients with DMD caused by mutations that are acquiescent to exon 53 skipping [19].

Milasen was developed in less than a year to treat Mila Makovec’s *CLN7* gene mutation leading to the development of Batten disease. This is a rare disease caused by one of at least 13 known mutations to the *CLN* gene that affects the cell’s ability to remove waste, such as excess proteins and lipids [115]. This disease is eventually fatal if left untreated. After genomic sequencing of Mila Makovec’s unique *CLN7* gene mutation, it was clear that this form of Batten disease was caused by improper exon splicing and the resulting premature translational termination [20]. Milasen targets a specific *CLN7* splice site, restoring proper splicing and function of CLN7. It eventually received FDA approval in 2018.

The FDA approved the use of casimersen in 2021 for the treatment of DMD caused by a mutation in the *DMD* gene that is amenable to exon 45 skipping [21]. Again, the mechanism of action of this drug is similar to that of golodirsen and eteplirsen.

#### 2.2.2. ASOs in Clinical Trials

ISS 1018 is in Phase II clinical trials for its synergistic effect with rituximab for the treatment of Non-Hodgkin’s lymphoma (NHL) [33]. NHL is a common cancer that begins in the lymphatic system and eventually spreads to other organs [116]. Rituximab has been extremely successful in treating many forms of NHL; however, there are still some forms that do not see any improvement following rituximab treatment. ISS 1018 was thought to have a beneficial effect when given to patients simultaneously with rituximab treatment. This drug can illicit immunostimulatory effects by signaling through the Toll-like receptor 9 and leads to proliferation and immunoglobulin production by B cells and the induction of tumor necrosis factor α (TNF-α), interleukin-12 (IL-12), interferon-α (IFN-α), and IFN-β by plasmacytoid dendritic cells [33]. The production of these cytokines triggers a powerful response in various other immune cell types that are not targeted directly by ISS 1018. Further, ISS 1018 can cause the maturation of dendritic cells that in turn cause an initiation of NK-cell and T-cell responses to tumor antigens [117,118]. To date, clinical trials have shown promise for 1018 ISS when used in conjunction with rituximab for the treatment of NHL [34].

Apatorsen (OGX-427) is in Phase I/II clinical trials for the treatment of castration-resistant prostate cancer (CRPC) or other metastatic cancers that have been demonstrated to express Hsp27, such as ovarian, breast, bladder, and non-small-cell lung cancers [35]. Apatorsen works by inhibiting the expression of Hsp27 by binding to and blocking translation of its mRNA [35]. Studies have shown effectiveness and tolerance of apatorsen in high doses in clinical trials thus far, with many patients showing decreased expression of cancer markers [35].

Phase II clinical trials of cenersen (EL625) have been conducted to test the efficacy of the drug for the treatment of acute myeloid leukemia [36]. Cenersen targets mutated p53, which is a proto-oncogene and leads to degradation of the p53 mRNA. This allows for cancer cells to respond to DNA damaging agents that once were not sensitive to such agents. Thus far, clinical trials have shown that patients that had not responded to standard chemotherapy or had relapsed shortly after standard chemotherapy had significantly better clinical outcomes following additional treatment with cenersen [36].

ARRx (AZD5312) has undergone Phase I/II testing for the treatment of CRPC [37]. ARRx was designed to target the androgen receptor (AR), which plays an important role in CRPC disease development and progression [119]. Preclinical trials have demonstrated the efficacy of this drug when used in conjunction with the pan-AKT inhibitor, AZD5363, for treating CRPC in a murine model [37].

Custirsen (OGX-011) is in Phase I/II clinical trials for treatment of advanced non-small-cell lung cancer that has previously been left untreated [38]. Custirsen targets the mRNA clusterin, which encodes for a chaperone protein that enables cell survival and causes resistance to various treatments [39]. Custirsen was shown to have minimal toxicity while significantly reducing the expression of clusterin in primary prostate tumors [38].

### 2.3. siRNA Therapeutics and Functional Implications

siRNAs are double-stranded RNAs that are relatively small (~21–25 nucleotides) and function to silence gene expression (Figure 1C) [120]. They occur naturally or can be chemically synthesized. Naturally occurring siRNAs originate from endogenous or viral precursor siRNAs. These precursors are roughly around 100 nucleotides long and are cleaved by Dicer into their mature siRNA structures [121]. Dicer leaves a 3′ overhang of two nucleotides that allows the siRNA to interact with the RISC complex, where it will initiate gene silencing [122]. Once it is bound, the RISC protein argonaute 2 (AGO) carries out cleavage of the sense strand [123]. This allows for the antisense strand to bind its target mRNA. Once the target RNA is bound to the antisense strand, its phosphodiester backbone is cleaved by AGO2. This leads to sequence-specific knockdown of the target mRNA and therefore causes gene silencing. Synthetic or naturally occurring siRNAs can therefore be used to knockdown the expression of a single protein coding gene. The use of the double-stranded siRNA to reduce expression of a target gene was first utilized in 1998 to target hlh-1, unc-54, unc-22, and fem1 in *Caenorhabditis elegans* (*C. elegans*) [1]. This groundbreaking work by Fire et al., eventually led to a Nobel prize award. Most importantly, they found that double-stranded RNAs (siRNAs) are more effective in downregulating their target mRNA than their single-stranded counterparts (ASOs) [1]. This highlights the advantage of siRNA technology over ASOs for the treatment of most human diseases.

#### 2.3.1. FDA-Approved siRNAs

The first siRNA therapeutic approved for use by the FDA was Patisiran [124]. It was approved in 2018 for the treatment of polyneuropathy caused by hereditary transthyretin-mediated (hATTR) amyloidosis [24]. hATTR amyloidosis is a genetic disorder that causes the buildup of abnormal TTR, which generally causes polyneuropathy when the build-up occurs in the peripheral nervous system [125]. Patisiran is an siRNA drug that targets the mutated *TTR* mRNA leading to mRNA degradation and decreased TTR protein expression [24]. This has been shown to greatly reduce TTR deposition in patients with polyneuropathy caused by hATTR amyloidosis [24].

Givosiran was the second FDA-approved siRNA therapeutic and is used to treat acute hepatic porphyria [126]. This disorder is caused by a plethora of deficiencies in enzymes involved in heme production and leads to a toxic buildup of porphobilinogen (PBG) and delta-aminolevulinic acid (ALA) [127]. Givosiran targets the mRNA of ALA synthase 1 in the liver and reduces the levels of disease-causing neurotoxic intermediates aminolevulinic acid and porphobilinogen [25].

Lumasiran was approved by the FDA for the treatment of primary hyperoxaluria type 1 (PH1) in 2020 [128]. Various mutations in the enzyme alanine-glyoxylate aminotransferase causes increased oxalate concentrations and calcium oxalate crystal formation leading to the development of PH1 [26]. Lumasiran targets the mRNA that encodes glycolate oxidase, leading to the depletion of the substrate for oxalate synthesis and sufficiently reducing oxalate concentrations [128].

In December 2021, inclisiran was approved by the FDA for the treatment of atherosclerotic cardiovascular disease (ASCVD) or heterozygous familial hypercholesterolemia (HeFH) (https://www.fda.gov/drugs/news-events-human-drugs/fda-approves-add-therapy-lower-cholesterol-among-certain-high-risk-adults (accessed on 30 December 2021)). These conditions are characterized by high LDL-C levels. Inclisiran works to lower LDL-C levels by targeting the mRNA encoding for proprotein convertase subtilsin/kexin type 9 (PCSK9), which is involved in lipid metabolism and the regulation of cholesterol levels [27]. Inclisiran has been shown to reduce LDL-C levels in patients that were unable to reduce these levels with statins alone [27]. Additionally, inclisiran has demonstrated increased efficacy in lowering LDL-C levels when administered in conjunction with statins in patients that statins alone have been partially effective in lowering LDL-C levels [27].

#### 2.3.2. siRNAs in Clinical Trials

TKM-080301 is in Phase I/II clinical trials for the treatment of hepatocellular carcinoma (HCC) [40]. HCC is typically characterized by the overexpression of Polo-like kinase 1 (PLK1) [129]. Therefore, targeting PLK1 may have beneficial effects for the treatment of HCC. To date, clinical trials have shown limited antitumor effects of TKM-080301 in patients with HCC [40].

Atu027 is in Phase I/II clinical trials for the treatment of advanced solid tumors and pancreatic adenocarcinoma [42,43]. It is designed to target the mRNA encoding protein kinase N3 (PKN3) in order to reduce the metastatic activity of tumors. Clinical trials have demonstrated the safety and efficacy of Atu027 in preventing adverse outcomes in patients with metastatic cancer [43].

siG12D LODER is in Phase I/IIa clinical trials for the treatment of pancreatic tumors in combination with chemotherapy [44]. It is a biodegradable implant containing a siRNA that targets the mRNA of the mutated *KRAS* oncogene, which can be surgically embedded in pancreatic tumors [45]. Mutated *KRAS* has been implicated in the development of most pancreatic cancers and is correlated with a worse prognosis for the patient [130]. Clinical studies have shown the potential efficacy of siG12D LODER in preventing tumor progression [44].

ARO-HIF2 is in Phase I clinical trials for the treatment of clear cell renal cell carcinoma (NCT04169711). This form of carcinoma is the most diagnosed form of kidney cancer [131]. In addition, it is associated with the inactivation of von Hippel–Lindau tumor-suppressor protein (pVHL) propelled by hypoxia-inducible factor 2 (HIF2) transcription factor deregulation [132]. Therefore, ARO-HIF2 aims to target the mRNA of HIF2 to inhibit tumor growth.

APN401 is currently in Phase I clinical trials to test its efficacy in treating patients with either metastatic or recurrent colorectal cancer, pancreatic cancer, or other solid tumors that are not surgically accessible [47]. It works by targeting casitas-B-lineage lymphoma protein-b (Cbl-b) that has been shown to limit lymphocyte activation [133]. Preclinical studies using murine tumor models demonstrated that Cbl-b inhibition enhances natural killer cell and T cell-mediated antitumor activity [133,134].

Vutrisiran (ALN-TTRSC02) is in Phase III clinical trials for the treatment of transthyretin (ATTR)-mediated amyloidosis with (NCT04153149) or without (NCT03759379) cardiomyopathy. ATTR-mediated amyloidosis is a condition caused by a buildup of TTR either caused by mutations in the *TTR* gene [48]. By targeting the *TTR* mRNA, vutrisiran is able to reduce TTR protein expression, leading to better outcomes in patients with ATTR-mediated amyloidosis [49].

### 2.4. miRNA Therapeutics and Functional Implications

miRNAs are non-coding RNAs that consist of ~20 nucleotides that are highly conserved between eukaryotic species. miRNAs were discovered in *C. elegans* by Ambros in 1993 [135]. That same year, Ruvkun found the first miRNA target genes [136]. The discovery that miRNAs can be used to downregulate target genes paved the way for miRNA therapeutics. miRNAs have excellent therapeutic potential due to their extraordinary targeting capability. For example, one miRNA can target anywhere from ten to hundreds of genes. Additionally, they tend to target multiple genes within the same pathway. miRNAs are naturally occurring molecules endogenous to our cells. Therefore, there is less chance for immunogenic response with miRNA therapeutics than their other synthetic RNA counterparts. Further, miRNA inhibitors and mimics can be used to restore or inhibit protein synthesis, respectively (Figure 1D).

Inhibition of protein synthesis or mRNA degradation is achieved through the miRNA associating with a variety of AGO proteins and modulating gene expression through the activity of the RISC complex [137]. When the miRNA is associated with AGO and the RISC complex is formed, the miRNA guides AGO to its target mRNA [138]. The seed sequence of the miRNA binds to the mRNA and causes either translational repression or mRNA degradation [139]. This leads to reduced target protein expression and therefore plays a key role in post-transcriptional gene regulation. This pathway can be enhanced by the supplementation of a miRNA mimic that is identical in sequence to the endogenous miRNA duplex. Restoring miRNA levels will lead to the repression of mRNAs that are overexpressed in certain conditions.

In contrast, restoration of protein synthesis is achieved by administering a miRNA inhibitor, a single-stranded miRNA that is complementary to a target miRNA. Once the miRNA inhibitor binds to the miRNA, it prevents the miRNA from associating with AGO. Therefore, miRNA inhibitors block the mRNA targeting ability of miRNAs and restore protein synthesis. This approach can be used to treat disorders caused by overexpression of a miRNA that leads to downregulation of certain disease preventing mRNAs.

#### miRNAs in Clinical Trials

While there are currently no miRNAs that are FDA approved, there are many in clinical development. For example, miravirsen has completed Phase II clinical trials for the treatment of Hepatitis (Hep) C [58]. Miravirsen is a miR-122 inhibitor that sequesters miR-122, which has been implicated in the promotion of the Hep C virus (HCV) life cycle [140]. Clinical trials to date have shown a significant reduction of HCV viral load in patients treated with miravirsen [58].

RG-012, also known as lademirsen, is in Phase II clinical trials for treatment of Alport syndrome (NCT02855268). This condition is caused by various mutations in the genes encoding collagen IV and leads to kidney disease as well as to both ocular and hearing deficiencies [141]. This syndrome is associated with increased levels of miR-21; therefore, it was hypothesized that a miR-21 inhibitor may work in treating this condition. Pre-clinical trials have demonstrated that a miR-21 inhibitor is extremely successful in preventing the onset of Alport syndrome, thus highlighting the therapeutic potential for this drug [59].

Cobomarsen is currently in clinical development for the treatment of various leukemias and lymphomas, such as adult T-cell leukemia/lymphoma (ATLL), chronic lymphocytic leukemia (CLL), the mycosis fungoides (MF) subtype of cutaneous T-cell lymphoma (CTCL), and the activated B-cell (ABC) subtype of diffuse large B-cell lymphoma (DLBCL) [142]. It works by targeting miR-155, which is associated with inflammation and the development of various leukemias and lymphomas [60,142,143,144]. Phase II clinical trials were initiated following successful Phase I trials (NCT02580552); however, they were terminated early due to business reasons unassociated with safety or efficacy (NCT03713320).

MRG-110 has completed Phase I clinical trials to test the safety efficacy of the miR-92a inhibitor in healthy patients (NCT03603431). Treatment with this inhibitor was found to significantly reduce miR-92a expression in these individuals when compared to patients treated with a placebo [145]. Additionally, increased expression of miR-92a has been associated with poor wound healing [61]. Further, miR-92a inhibition has been shown to improve wound healing in vivo in preclinical models [146]. Therefore, MRG-110 may be effective in treating impaired wound healing in conditions such as diabetes.

RG-125 (AZD4076) completed Phase I/IIa clinical trials for the treatment of Type 2 Diabetes (T2D) and Non-Alcoholic Fatty Liver Disease (NAFLD) (NCT02826525). This drug inhibits miR-103/107, in which overexpression of these miRNAs has been shown to correlate with the development of T2D and NAFLD [147]. Further, preclinical studies have demonstrated that a miR-103/107 inhibitor can improve insulin sensitivity in obese mice [62]. This along with successful Phase I/IIa clinical trials emphasizes the therapeutic potential of miR-103/107 inhibitors for the treatment of T2D and NAFLD.

RGLS4326 completed Phase 1b clinical trials for the treatment of autosomal dominant polycystic kidney disease (ADPKD) (NCT04536688). This disease is caused by mutations in *PKD1* and *PKD2*, resulting in decreased expression of PC1 and PC2 [148]. miR-17 has been shown to bind and downregulate *PKD1* and *PKD2* gene expression, while miR-17 inhibitors have been shown to restore *PKD1* and *PKD2* expression [63]. Finally, inhibition of miR-17 by RGLS4326 in humans was also shown to significantly increase PC1 and PC2 levels in patients with ADPKD (NCT04536688).

CDR132L has completed Phase I clinical trials for the treatment of heart failure of ischemic origin (NCT04045405). Hypertrophy of cardiomyocytes has been shown to be caused by overexpression of miR-212/132 family, leading to heart failure [149]. Further, preclinical studies have shown that inhibiting miR-132 can improve heart function in animal models of heart failure [150,151]. Finally, initial clinical studies have shown exceptional efficacy of CDR132L for treating patients with heart failure (NCT04045405).

TargomiRs has been studied as either a second- or third-line treatment for recurrent malignant pleural mesothelioma and non-small-cell lung cancer. Downregulation of miR-16 has been implicated in the development of many types of cancer, such as chronic lymphocytic leukemia and non-small-cell lung cancer [152,153]. Additionally, miR-16 mimics act to increase miR-16 levels in order to target and downregulate multiple oncogenes and lead to tumor regression [154]. TargomiRs, a miR-16 mimic, has shown substantial preclinical efficacy in the treatment of many types of cancer [155,156]. Finally, initial clinical trials have shown that TargomiRs has antitumor effects in patients with malignant pleural mesothelioma [65].

Remlarsen has completed Phase II clinical trials for the treatment of keloid scars. Keloid scars are caused by a fibroproliferative disorder that causes excess production of extracellular matrix proteins and collagen [157]. miR-29 has been shown to negatively regulate multiple genes involved in the fibrotic response [158,159] and therefore reduces fibrosis [160]. Further, remlarsen, a miR-29 mimic, has shown a significant reduction in collagen expression and fibrosis in skin wounds [161]. Therefore, remlarsen may have therapeutic potential in treating keloid scars as well as scleroderma.

There have been some miRNA drug candidates that have demonstrated severe adverse effects. For example, MRX34 is a miR-34 mimic that showed success in preclinical trials for the treatment of cancer [162,163,164]. Expression of miR-34 has been shown to be significantly reduced in many different types of cancer [165,166,167]. Further, miR-34 targets many different oncogenes and therefore can theoretically hamper tumor progression [168,169,170]. While early Phase I clinical trials were successful in reducing the miR-34a target oncogenes in a dose-dependent manner [67,171], they were eventually halted after several patients had severe adverse reactions to treatment [171].

Finally, there are multiple miRNA therapeutics that have demonstrated substantial preclinical efficacy for various disorders. For example, miR-10b-5p is in preclinical development for the treatment of diabetes and associated gut motility disorders [13]. Additionally, a miR-101-3p inhibitor in combination with chemotherapeutic agents has been shown to effectively reduce CRC cell proliferation [172]. Further, miR-221 is another possible target for many different types of cancer, as its expression is increased in glioblastoma, osteosarcoma, CRC, etc. [173,174,175]. The therapeutic potential of miRNAs is limitless and will likely lead to improved treatment options for many different diseases, particularly ones with multiple underlying pathophysiological mechanisms.

### 2.5. Aptamer Therapeutics and Functional Implications

Aptamers are single-stranded RNA, DNA, or RNA-DNA hybrids that have often been classified as chemical antibodies. They often are around 20–100 base pairs long and fold into specific tertiary structures that allow them to specifically bind to their respective targets (Figure 1E) [176]. Aptamers can be designed to target carbohydrates, peptides, proteins, and various other molecules, making them an attractive therapeutic option for various diseases.

Aptamers are generated using the systematic evolution of ligands by exponential enrichment (SELEX) method. This procedure uses a randomized library that contains ~4^# of nucleotides^ individual sequences that can be tested simultaneously [177]. While aptamers have been synthesized containing 8–228 nucleotides, most are around 20 nucleotides long [178]. This large library allows for trillions of sequences to be tested to find ones that are able to bind the target molecule. These sequences then continue on to further rounds of selection, thus increasing the population of aptamers that are able to bind the target with high affinity. Eventually, there are specific sequences that dominate the population of library species. This process is extremely fast in comparison to traditional peptide synthesis strategies.

Aptamers can act through three main mechanisms of action: (1) aptamers that are specific to a particular cell type can deliver other therapeutic agents to the target tissue or cells; (2) aptamers can act as an agonist and thus functionally activate their target molecules; (3) aptamers work as antagonists and block the interaction of molecules in pathways associated with disease development [176].

#### 2.5.1. FDA-Approved Aptamers

Pegaptanib was the first-ever FDA-approved aptamer and is used for the treatment of neovascular age-related macular degeneration. This disease is characterized by retinal degeneration, causing vision loss [179]. Increased vascular endothelial growth factor (VEGF) has been associated with this condition [180]. Therefore, anti-VEGF treatment was thought to be an efficient method for treating this disease. Pegaptanib was found to have high affinity for VEGF and caused it to be sequestered, preventing it from binding to its receptor. After successful clinical trials showing that pegaptanib improved or halted vision loss [28], it was approved for use by the FDA in 2004.

Defibrotide was approved by the FDA in 2020 for the treatment of hepatic veno-occlusive disease/sinusoidal obstruction syndrome [29]. This can be a life-threatening complication caused by chemotherapy and hematopoietic stem cell transplant (HSCT) conditioning [181]. Defibrotide has been reported to stabilize endothelial cells via reduced endothelial cell activation [182]. This protects endothelial cells from further damage and rescues this condition. Both FDA-approved medicines have minimal side effects and highlight the promise of aptamer-based therapeutics.

#### 2.5.2. Aptamers in Clinical Trials

NOX-A12 is an RNA aptamer that is in clinical trials for the treatment of various types of cancers, for example, pancreatic cancer, colorectal cancer, and multiple myeloma [50,183,184]. This drug works by neutralizing CXCL12, which leads to an increase in circulating tumor-infiltrating T-cells [185]. To date, clinical trials have demonstrated the potential of NOX-A12 in the treatment of various types of cancer [50,183,184].

NOX-E36 has completed Phase I clinical trials for the treatment of diabetes and albuminuria (NCT01547897). Increased HbA1c and albumin/creatinine ratio (ACR) are hallmarks of these conditions. NOX-E36 has been shown to reduce these levels in patients with diabetes and albuminuria [51] by binding to CCL2, a pro-inflammatory cytokine, with high affinity [186]. Therefore, this therapeutic drug may have beneficial effects on the treatment of diabetes and related conditions.

There are other aptamers in clinical trials for various human diseases that are composed of DNA. For example, AS1411 is a DNA aptamer for the treatment of cancers such as acute myeloid leukemia [187]. However, since the purpose of this review is to highlight the potential of RNA therapeutics, we will not discuss these aptamers in detail.

## 3. Conclusions and Future Perspectives

RNA molecules are multi-functional and are extremely versatile. State-of-the-art studies have demonstrated considerable promise for the clinical use of RNA therapeutics to treat and prevent human diseases. Further, RNA therapeutics are relatively cheaper, easier, and faster to develop than traditional protein- and small molecule-based drugs. RNA therapeutic approaches vary in how they treat different clinical conditions. For example, siRNAs are highly specific with only one mRNA target; therefore, they are good for the treatment of diseases where pathologies are caused by the alternations of only one single gene. However, miRNAs have the virtue of targeting multiple mRNAs; consequently, they are suitable for the treatment of diseases in which various pathologies and/or alternations of many genes are involved. The current challenges for RNA therapeutics include: (i) Cell specificity—ideally, the best RNA therapeutic molecule would have on-target cell specificity without off-target and undesired on-target effects. (ii) Cell-specific delivery agents—one of the most significant challenges in RNA therapeutics is efficient and stable delivery of the molecule to the cell type of interest and being functionally active to perform their role.

Clinical trials should focus heavily on early study design to prevent possible adverse outcomes such as acute toxicity. Additionally, this early study design should focus on in vivo functional assays rather than in vitro functional assays alone. Further, the most critical step in RNA therapeutics development is to compare the clinical outcomes in their ability to fix the mechanistic parameters. To accomplish this goal, the RNA therapeutic candidates must be rigorously examined, particularly for their immune tolerance, pharmacokinetics, and pharmacodynamics. However, RNA therapeutic agents are likely developed based on the cellular and molecular mechanisms underlying pathologies of the diseases; thus, these molecules are placed in a prime position for future clinical trials. The current knowledge gaps warrant a modern approach to better understand the pathologies at the cellular and molecular level that will enable us to tackle the therapeutic approaches to treat the disease, not only improving the symptomology, but also fixing the exact cause.

While there are currently challenges to RNA therapeutic development, unprecedented interdisciplinary approaches and promising developments in modern science, along with improved early study design for clinical trials, will overcome these obstacles in the foreseeable future. This will provide substantial hope for the clinical utility of RNA therapeutics for different disease conditions and lead to a better quality of life for millions of patients.

## Figures and Tables

**Table 1:** FDA-approved RNA therapeutics in clinical care.

Product	Route of Delivery	Target	Mechanism of Action	Disease/Clinical Outcome	Company	Approval Status	References
***ASO***							
Fomivirsen	IVT	CMV mRNA	Downregulates IE2	Cytomegalovirus (CMV) retinitis	Ionis Pharmaceutical, Novartis	FDA (1998)	[14]
Mipomersen	SC	apo-B-100 mRNA	Downregulates ApoB	Homozygous familial hypercholesterolemia	Kastle Therapeutics, Ionis Pharmaceuticals, Genzyme	FDA (2013)	[15]
Nusinersen	ITH	SMN2 pre-mRNA	Splicing modulation	Spinal muscular atrophy	Ionis Pharmaceuticals, Biogen	FDA (2016)	[16]
Eteplirsen	IV	Exon 51 of DMD	Splicing modulation	Duchenne muscular dystrophy	Sarepta Therapeutics	FDA (2016)	[17]
Inotersen	SC	TTR mRNA	Downregulates transthyretin mRNA	Familial amyloid polyneuropathy	Ionis Pharmaceuticals	FDA (2018)	[18]
Golodirsen	IV	Exon 53 of DMD	Splicing modulation	Duchenne muscular dystrophy	Sarepta Therapeutics	FDA (2019)	[19]
Milasen	Intrathecal	CLN7	Splicing modulation	Mila Makovec’s *CLN7* gene associated with Batten disease	Boston Children’s Hospital	FDA (2018)	[20]
Casimersen	IV	Exon 45 of DMD	Splicing modulation	Duchenne muscular dystrophy	Sarepta Therapeutics	FDA (2021)	[21,22,23]
***siRNA***							
Patisiran	IV	TTR mRNA	Downregulation of transthyretin	Polyneuropathy caused by hATTR amyloidosis	Alnylam	FDA (2018)	[24]
Givosiran	SC	ALS1 mRNA	Downregulation of ALAS1	Acute hepatic porphyria	Alnylam	FDA (2020)	[25]
Lumasiran	SC	HAO1 mRNA	Downregulation of glycolate oxidase	Primary hyperoxaluria type 1	Alnylam	FDA (2020)	[26]
Inclisiran	SC	PCSK9	Downregulation of proprotein convertase subtilsin/kexin type 9	Atherosclerotic cardiovascular disease	Novartis	FDA (2021)	[27]
***Aptamer***							
Pegaptanib	Intravitreal	Heparin-binding domain of VEGF-165	Blocking VEGF-165	Neovascular age-related macular degeneration	OSI Pharmaceuticals	FDA (2004)	[28]
Defibrotide	IV	Adenosine A1/A2receptor	Activating Adenosine A1/A2 receptor	Veno-occlusive disease in liver	Jazz Pharmaceuticals	FDA (2020)	[29,30]
***mRNA***							
BNT162b2	IM	Immunogenicity and antibody response to SARS-CoV-2 S antigens	SARS-CoV-2 S antigens’ expression	COVID-19	BioNTech and Pfizer	FDA (2020)	[31]
mRNA-1273	IM	Immunogenicity and antibody response to SARS-CoV-2 S antigens	SARS-CoV-2 S antigens’ expression	COVID-19	Moderna	FDA (2020)	[32]

**Table 2:** RNA therapeutics in clinical development.

Oligonucleotide Therapeutics	Route of Delivery	Target	Mechanism of Action	Disease/Clinical Outcome	Company	Clinical Trial Status	References
***ASO***							
1018 ISS	IV	TLR9	Enhancement of cytotoxic effector mechanisms	Non-Hodgkin’s Lymphoma	Dana-Farber Cancer Institute, Brigham and Women’s Hospital, Massachusetts General Hospital, University of Rochester	NCT00251394 (Phase II)	[33,34]
Apatorsen (OGX-427)	IV	HSP27	Inhibits expression of heat shock protein (Hsp27)	Urologic Cancer, Bladder Cancer, Prostate Cancer, Urothelial Cancer, Non-Small-Cell Lung Cancer	Achieve Life Sciences PRA Health Sciences	NCT00487786, NCT01454089 (Phase I/II)	[35]
Cenersen (EL625)	IV	TP53	Blocks the effects of p53	Acute Myelogenous Leukemia, Lymphoma	Eleos, Inc.	NCT00074737 (Phase II)	[36]
ARRx (AZD5312)	IV	AR	Suppression of human AR expression	Prostate Cancer	AstraZeneca	NCT02144051, (Phase I/II)	[37]
Custirsen (OGX-011)	IV	ApoJ	Inhibition of clusterin expression	Prostate Cancer, Breast Cancer, Non-Small-Cell Lung Cancer	NCIC Clinical Trials Group, Achieve Life Sciences	NCT00054106, NCT00138658, (Phase I/II)	[38,39]
***siRNA***							
TKM-080301	Intra-arterial/IV	PLK1	Inhibition of PLK1 activity	Cancer with hepatic metastases, Hepatocellular Cancer	National Cancer Institute, Arbutus Biopharma Corporation	NCT01437007, NCT02191878, (Phase I/II)	[40,41]
Atu027	IV	PNK3	Silences expression of PNK3	Solid Tumors, Pancreatic Cancer	Silence Therapeutics GmbH, Granzer Regulatory Consulting & Services	NCT00938574, NCT01808638 (Phase I/II)	[42,43]
siG12D LODER	Locally implanted through EUS biopsy procedure	KRASG12D	Inhibits KRAS expression	Pancreatic Cancer	Silenseed Ltd.	NCT01676259, NCT01188785 (Phase I/II)	[44,45]
ARO-HIF2	IV	HIF2A	Deregulation of HIF2A	Clear Cell Renal Cell Carcinoma	Arrowhead Pharmaceuticals	NCT04169711 (Phase I)	[46]
APN401	IV	CBLB	Inhibition of Cbl-b enhances natural killer cell and T cell mediated antitumor activity	Brain Cancer, Melanoma, Pancreatic Cancer, Renal Cell Cancer	Wake Forest University Health Sciences, National Cancer Institute	NCT03087591, NCT02166255 (Phase I)	[47]
Vutrisiran	SQ	TTR	Reduces TTR protein expression	Transthyretin mediated amyloidosis with or without cardiomyopathy	Alnylam Pharmaceuticals	NCT03759379 NCT04153149 (Phase 3)	[48,49]
***Aptamer***							
NOX-A12	IV	CXCL12	Disrupts CXCR4-CXCL12 interactions	Pancreatic Cancer, Colorectal Cancer, Multiple myeloma	NOXXON Pharma AG, Merck Sharp & Dohme Corp.	NCT01521533, NCT01521533, NCT03168139 (Phase I/II)	[50]
NOX-E36	IV/SQ	CCL2	Specifically binds and inhibits the pro-inflammatory chemokine CCL2	Diabetic nephropathy	NOXXON Pharma AG	Phase I	[51]
***mRNA***							
CVnCoV	IM	Immunogenicity and antibody response to SARS-CoV-2 S antigens	SARS-CoV-2 S antigens’ expression	COVID-19	CureVac AG	NCT04652102 (Phase III)	[52]
AZD8601	Epicardial	VEGF-A	Restores VEGF-A expression	Ischemic heart disease	AstraZeneca	NCT03370887 (Phase II)	[53]
MRT5005	Inhalation	CFTR	Restores CFTR expression	Cystic Fibrosis	Translate Bio	NCT03375047 (Phase I/II)	[54]
mRNA-3704	IV	MUT	Restores MUT expression	Methylmalonic aciduria	Moderna	NCT03810690 (Phase I/II)	[55,56]
BNT111	IV	Targets four non-mutated, TAAs (NY-ESO-1, MAGEA3, tyrosinase and TPTE	Induction of immune response against the four selected malignant melanoma-associated antigens (New York-ESO 1 (NY-ESO-1), tyrosinase, Melanoma-associated antigen A3 (MAGE-A3), and Trans-membrane phosphatase with tensin homology (TPTE))	Advanced Melanoma	BioNTech SE	NCT02410733 (Phase I)	[57]
***miRNA***							
Miravirsen	SC	miR-122	miRNA-inhibitor	HCV	Roche/Santaris	NCT01200420 (Phase II)	[58]
RG-012 (lademirsen)	SC	miR-21	miRNA-inhibitor	Alport Syndrome	Sanofi	NCT03373786 (Phase II)	[59]
Cobomarsen	IV/SQ	miR-155	miRNA-inhibitor	Cutaneous T-Cell Lymphoma/Mycosis Fungoides	miRagen	NCT03713320, NCT02580552 (Phase II)	[60]
MRG-110	Intradermal	miR-92a	miRNA-inhibitor	Wound healing	miRagen	NCT03603431 (Phase I)	[61]
AZD4076	SC	miR-103/107	miRNA-inhibitor	T2D with NAFLD	AstraZeneca	NCT02826525 (Phase I/IIa)	[62]
RGLS4326	SC	miR-17	miRNA-inhibitor	Autosomal dominant polycystic kidney disease	Regulus Therapeutics Inc.	NCT04536688 (Phase I)	[63]
CDR132L	IV	miR-132	miRNA-inhibitor	Heart Failure	Cardior Pharmaceuticals GmbH	NCT04045405 (Phase I)	[64]
TargomiRs	IV	miR-16	miRNA-mimic	Malignant Pleural Mesothelioma	EnGeneIC Limited	NCT02369198 (Phase I)	[65]
Remlarsen	Intradermal	miR-29	miRNA-mimic	Keloids, scleroderma	miRagen	NCT03601052 (Phase II)	[66]
MRX34	IV	miR-34a	miRNA-mimic	Melanoma	miRNA Therapeutics, Inc.	NCT01829971 (Phase I)	[67]
